# The Effectiveness of Laser Peripheral Iridotomy in Adolescent Eyes with Ocular Hypertension and Concave Configuration of the Peripheral Iris

**DOI:** 10.1155/2022/4068026

**Published:** 2022-02-28

**Authors:** Alina Bakunowicz-Łazarczyk, Beata Urban, Małgorzata Krętowska

**Affiliations:** ^1^Department of Paediatric Ophthalmology and Strabismus, Medical University of Bialystok, Waszyngtona 17, Bialystok 15-274, Poland; ^2^Faculty of Computer Science, Bialystok University of Technology, Wiejska 45A, Białystok 15-351, Poland

## Abstract

**Purpose:**

To evaluate the efficacy of laser peripheral iridotomy (LPI) in preventing deterioration in eyes with ocular hypertension (OHT) and concave configuration of the iris.

**Materials and Methods:**

This was a retrospective study, which was carried out within a period of 3–5 years. Twenty-four patients with OHT and concave irises were treated with LPI and followed up periodically. IOP, central corneal thickness (CCT), anterior chamber depth (ACD), scleral spur angle (SSA), global neuroretinal rim (NRR) thickness, and global retinal nerve fiber layer (RNFL) were examined before and after LPI.

**Results:**

The average age of the 24 patients was 14.21 ± 1.41 (13–17.5) years on admission. The initial IOP of the 48 eyes was 23.21 ± 1.56 mmHg in RE and 22.96 ± 2.1 mmHg in LE before LPI. All 48 eyes had concave irises in both eyes. All eyes treated with LPI have shown iris flattening, which has persisted during follow-up (mean 1.54 ± 0.9 years). At the last follow-up visit, the average IOP was 17.58 ± 2.63 (14–21) mmHg in RE and 17.58 ± 2.86 (14–21) mmHg in LE, which was statistically lower than that of the baseline (*p* < 0.001). There were significant changes in SSA in both eyes and global RNFL in RE after LPI.

**Conclusions:**

In the current study, LPI resulted in an IOP-lowering effect and iris flattening in adolescent eyes with a concave configuration of the peripheral iris.

## 1. Introduction

The pigment dispersion syndrome (PDS) is characterized by structural changes in the anterior segment, especially a structural disturbance in the iris pigment epithelium, that lead to the shedding of the pigment from the posterior surface of the iris into the anterior segment and its deposition on various ocular structures [1, 2]. In PDS, the iris has a concave configuration and is often inserted into the posterior ciliary body band. Anomalous iridozonular contact leads to rubbing of the pigmented iris epithelium against the zonular fibers and pigment release throughout the anterior segment [3, 4]. Pigment dispersion results in an accumulation of pigment granules within the aqueous humor and the outflow tissue. PDS can lead to a secondary elevation of IOP and cause PG. Other clinical presentations of PDS include Krukenberg spindle, radial iris transillumination defects, diffuse pigmentation of the anterior chamber angle, Sampaolesi-like line, Scheie/Zentmayer stripe, and sometimes pigment deposition on the posterior lens capsule [1–4]. When IOP in patients with PDS is high or when signs of glaucomatous optic nerve develop, treatment should be initiated. Topical antiglaucoma medications are the first choice. Sometimes, laser procedures are considered. However, their current role is still unclear. Laser peripheral iridotomy (LPI)—an alternate treatment of elevated IOP—is one such method. This procedure creates an opening in the iris tissue and inhibits pigment release.

Ocular hypertension (OHT) is defined as the presence of intraocular pressure higher than 21 mmHg, with no optic nerve damage or visual field loss. OHT is considered the most important risk factor for glaucoma. Without proper intervention, over 10% of the patients with ocular hypertension would develop glaucoma in the following 5 to 10 years [5]. Therefore, lowering IOP is the main strategy for preventing glaucoma in patients who are at risk. Regrettably, patients with OHT usually have none or very few ocular symptoms and little disturbance in visual acuity, making its diagnosis and treatment a huge challenge [6]. In addition, there is no consensus concerning the management of OHT.

The aim of this study was to assess the effects of LPI on lowering IOP in adolescents with OHT and concave configuration of the iris visible in anterior segment optical coherence tomography (AS-OCT). We assumed that peripheral iris concavity may be a risk factor for the development of PDS in adolescents, making pigment release easier. We also wanted to investigate whether laser therapy results in any changes in the anterior segment parameters and in the optic disc and retinal parameters, as assessed by optical coherence tomography.

## 2. Materials and Methods

Twenty-four patients with ocular hypertension and concave configuration of the iris were treated with laser peripheral iridotomy and followed up periodically in the Department of Paediatric Ophthalmology and Strabismus. None of the patients had PDS. Inclusion criteria were as follows: IOP >21 mmHg, age between 12 and 18 years, no signs of glaucoma, best-corrected visual acuity 1.0, no history of ocular or systemic disorders, and good quality of OCT images. The exclusion criterion was peripapillary atrophy. All patients underwent a full-eye examination, including best-corrected Snellen visual acuity, IOP measurement by TonoPen, visual field, corneal pachymetry, slit-lamp examination, and stereoscopic optic disc examination with a 78-diopter lens, gonioscopy, AS-OCT, and SLO/OCT Spectralis imaging.

**Figure 1:**
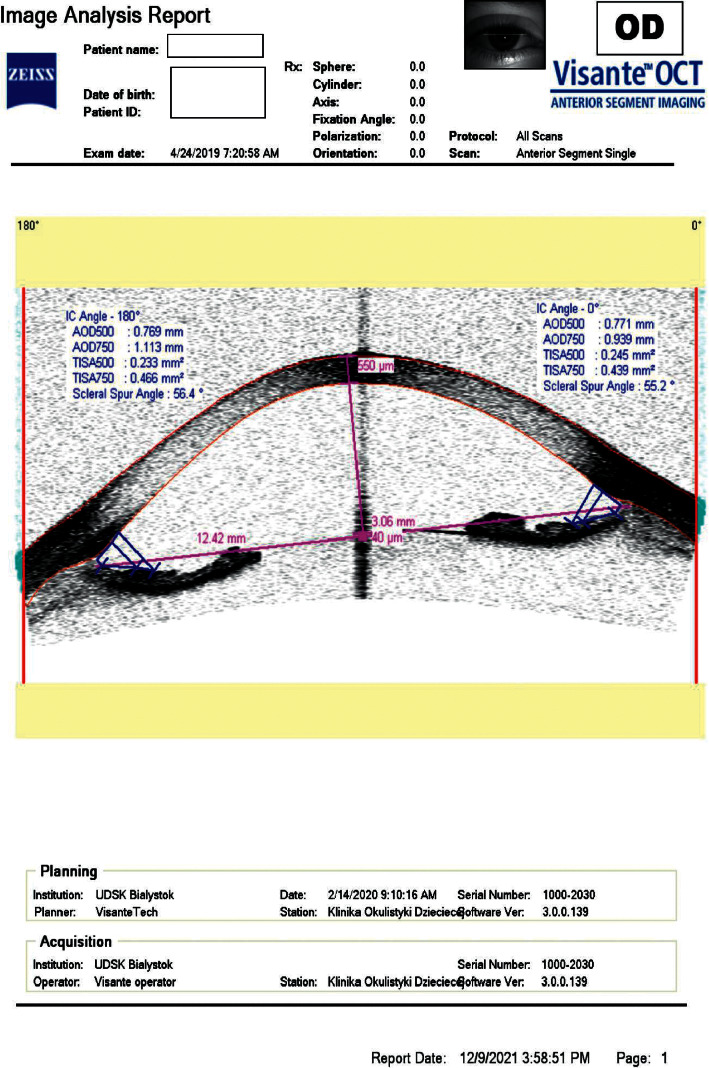
Concave configuration of the iris before LPI.

**Figure 2:**
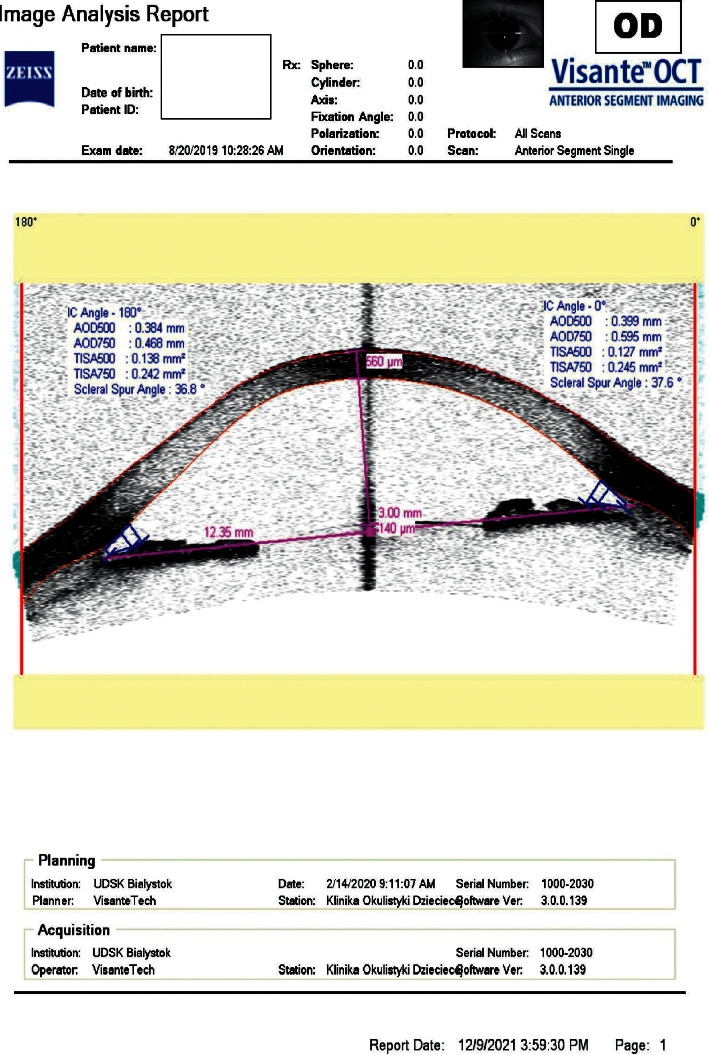
Flattening of the iris after LPI.

The main items of investigation included evaluation of IOP and selected parameters of the anterior and posterior segments before and 2 months after laser treatment. To examine the iris configuration, central corneal thickness (CCT), anterior chamber depth (the distance between the anterior surface of the cornea and the anterior border of the lens capsule (ACD)), and scleral spur angle (SSA, a measurement of the angle formed at the apex of the iris recess), with the arms of the angle passing through the angle-opening distance/AOD/at 500 *µ*m anterior to the scleral spur/AOD500 line/) in the nasal and temporal quadrants, all subjects underwent anterior segment optical coherence tomography (AS-OCT Visante, Carl Zeiss Meditec AG, Oberkochen, Deutschland) (Figures 1 and 2).

The AS-OCT was performed by an ophthalmic imaging technician. To perform the global neuroretinal rim (NRR, the neuroretinal tissue between the optic disc margin and the cup margin around the entire circumference of the optic nerve head) thickness and global retinal nerve fiber layer thickness (RNFL) measurements, optical coherence tomography (SLO/OCT Spectralis) has been used by the same ophthalmic imaging technician. The decision on whether LPI was clinically indicated was made by the ophthalmologist based on the AS-OCT images obtained during the first visit.

The LPI was performed using the Laser LightLas SLT/YAG/577 (LightMed). All eyes were instilled with 2% pilocarpine prior. The LPI was placed in the superior region (between 11 and 1 o'clock) of the iris, as peripherally as possible, by the same ophthalmologist, and 8–12 shots of 3.0–3.5 mJ power were applied. After the procedure, patients were given a 5-day course of topical loteprednol etabonate 0.5% to relieve postlaser inflammation. At the last follow-up visit, AS-OCT was repeated for each patient to evaluate the iris configuration.

### 2.1. Statistical Analysis

The Shapiro–Wilk test was used to verify the hypotheses of normal distribution of the analyzed parameters. For comparing the parameter values before and after the treatment, the paired *t*-test and Wilcoxon signed-rank tests were applied. In addition, Pearson's rank correlation coefficient was used as a correlation measure between variables. The unpaired *t*-test was used to compare the two groups of patients. The Holm–Bonferroni method was applied for multiple comparisons. In all the comparisons, a 0.05 significance level was applied.

### 2.2. Ethical Issues

The study was conducted according to the tenets of the Declaration of Helsinki and had received approval from the Local Ethics Committee. All parents signed a consent form before the inclusion in the study.

## 3. Results

The study included 48 eyes of 24 patients with OHT. The average age of adolescents (14 boys and 10 girls) was 14.21 ± 1.41 years (13–17.5 years) on admission. All patients had best-corrected visual acuity of 1.0. Fourteen adolescents had myopia (4 cases-mild myopia, 2 cases-moderate myopia, and 8 cases-high myopia), and 10 people were emmetropic. The initial IOP of the 48 eyes was 23.21 ± 1.56 mmHg in RE and 22.96 ± 2.1 mmHg in LE before LPI. In addition, all 48 eyes had a concave configuration of the iris in AS-OCT. The selected examined parameters of the anterior and posterior segments are presented in Table 1. The iris became flat in all treated eyes after the laser treatment. There were no adverse postlaser complications. At the last follow-up visit, the average IOP was 17.58 ± 2.64 (14–21) mmHg in RE and 17.58 ± 2.86 mmHg (14–21) in LE, which was statistically lower than that of baseline (*p* < 0.001 and *p* < 0.001, respectively; Table 2).

There were statistically significant differences in SSA in the nasal and temporal quadrants in both the eyes before and after the laser treatment (Table 1). There were no essential changes in CCT and ACD in both the eyes after the laser procedure.

Apart from the global retinal nerve fiber layer (RNFL) thickness in RE (*p*=0.003), differences in global RNFL in LE and global neuroretinal rim (NRR) thickness in both eyes on OCT before and after LPI were not significant (Table 3).

We analyzed the effect of gender, age, and observation time on the intraocular pressure reduction after LPI. There were no differences in IOP reduction after LPI depending on gender (Table 4). There was also no correlation between age and IOP reduction after LPI (Table 5). We omitted the impact of myopia presence on the results of LPI due to limited sample size.

## 4. Discussion

Reverse pupillary block has been considered as one possible pathogenetic mechanism for backward bowing of the iris, leading to iris-zonular rubbing and distribution of melanin granules in the anterior chamber in PDS [1, 3, 7]. Karickhoff was the first to suggest that LPI may relieve the posterior bowing of the peripheral iris by equalizing the pressure between the anterior and posterior chambers [4]. LPI is an alternative treatment to medications, because it can reverse backward bowing of the iris and thus may prevent further melanin dispersion and development of pigmentary glaucoma. The effect of Nd:YAG laser iridotomy as a prophylactic and potentially causal treatment in PDS can be effortlessly visualized by OCT [8]. However, the usability of LPI has not been completely established in both PDS and PG. Besides, nearly all publications are concerned with adult patients. Areaux and Grajewski reported the clinical and ultrasound biomicroscopic findings of PDS in a 14-year-old girl with Marfan syndrome and its favorable response to bilateral LPI [9].

In the present study, we have evaluated the effect of LPI in adolescents with ocular hypertension and a concave configuration of the iris visualized by optical coherence tomography. We assumed that the concave iris may contribute to the development of PDS, especially in myopic adolescents. Therefore, making the hole in the iris will protect from IOP elevation by relieving iridozonular contact and diminishing pigment release. We are conscious that although laser iridotomy produces a planar iris configuration, some eyes may retain a concave iris configuration. Iris flattening and a decrease in IOP were observed in all eyes treated with LPI. IOP was reduced by 5.5 mmHg after LPI with a mean follow-up of 1.54 ± 0.9 years. The mean decline after laser treatment was 5.6 mmHg in the right eyes and 5.4 mmHg in the left eyes.

Gandolfi and Vecchi have proved that YAG laser iridotomy may reduce the incidence of ocular hypertension in eyes affected by PDS. In their opinion, this effect, being less pronounced after 40 years of age, may be of clinical relevance in young subjects [10]. Similar results were obtained by Qing et al., who noted that LPI effectively prevents progression in eyes with PDS [11]. However, at the end of the 10-year follow-up, approximately one-third of the whole PDS patient population treated with LPI showed an IOP increase of 5 mmHg or higher in at least 1 eye [12]. Similar observations were made by Scott et al., who showed that there was no benefit of LPI in preventing progression from PDS with ocular hypertension to pigmentary glaucoma within 3 years of follow-up [13]. The lack of reduction in IOP after LPI in patients with PDS/PG may be explained by the fact that this procedure cannot correct structural abnormalities or changes in trabecular meshwork. Besides, among five trials assessing the effectiveness of LPI, no clear benefit was reported for this procedure compared with no laser in eyes with PG for visual field loss or PDS as regards preventing visual field progression [14].

We have observed an increase in ACD and a decrease in CCT in both eyes after LPI, but these changes were not statistically significant (Table 1). In the current study, the mean CCT was 548 ± 43 *µ*m in the right eyes and 552 ± 42 *µ*m in the left eyes, so the corneas were rather thin. The ophthalmologist should consider thin corneal thickness measurements as one of the key risk factors for developing glaucoma. The Ocular Hypertension Treatment Study (OHTS) determined that a thin cornea is a very important factor that can predict the transformation of ocular hypertension in primary open-angle glaucoma [15]. Five-year follow-up has shown that patients with CCT lower than 555 *µ*m had a 3 times higher risk of developing the disease in comparison with a subject with a CCT higher than 588 *µ*m. This could mean the possibility that some of our patients have an additionally increased risk of developing POAG in the future.

In the current study, we observed the significant reduction of SSA as the result of iris flattening (Table 1). SSA in both nasal and temporal quadrants were significantly smaller after LPI. This post-LPI narrowing of the scleral spur angles may be explained by the iris flattening. In the current study, we observed the iris flattening in all eyes after the laser treatment and it persisted during the last follow-up (mean 1.54 ± 0.9 years). Aptel et al. evaluated anterior chamber volume, iris volume, and iridolenticular contact area before and after LPI in eyes with PDS using AS-OCT [16]. After estimating the biometric parameters, they concluded that PDS eyes show little resistance to an iris that is stretched and pushed against the lens when there is a pressure difference across the iris. It is possible to assume that all eyes of our patients have a normal structure of iris, which responds to LPI with flattening, leading to significant lowering of IOP.

The assessment of circumpapillary RNFL thickness serves as an important tool in the diagnosis and follow-up of glaucoma. SD-OCT is one of the imaging modalities that is most often used worldwide to evaluate the optic nerve head and the neuroretinal rim. We have observed that the flattening of the iris in examined eyes was associated with both a lowering of intraocular pressure and an improvement in both global RNFL thickness and global NRR thickness in both eyes after the laser treatment, but only changes in RNFL in RE were significant (Table 3). In the current study, the mean peripapillary RNFL thickness was 94.63 ± 10.46 *µ*m in the right eyes and 95.54 ± 9.6 *µ*m in the left eyes and this corresponds with RNFL values reported in other studies [17–19]. To our astonishment, we observed 2 *µ*m RNFL thickening in the right eyes (*p*=0.003) and 2.42 *µ*m RNFL thickening in the left eyes (NS) after LPI. One of the explanations may be the decentration of the circle scan during OCT—its displacement by just 0.1 mm can result in a 2.3 ± 2.0 *μ*m error in average RNFL thickness [20]. It is also possible that ageing itself may be the reason for these changes in the paediatric population. Another reason is the fact that RNFL mainly consists of the axons of the retinal ganglion cells, but it is also composed of glial cells and blood vessels [21]. Numerous studies have shown that ocular blood flow increases after a significant decline in IOP [22, 23]. In the study of Ch'ng at al., peripapillary RNFL thickness was transiently increased in a total of 40 eyes 1 month after glaucoma surgery, but in their opinion, RNFL thickness was completely IOP independent [23]. Our patients were adolescents, and we can theoretically assume that perhaps the lowering of IOP after LPI increased their ocular blood flow and it could thus contribute to RNFL thickening. Certainly, further research is essential for the evaluation of ocular blood flow in OHT using OCTA.

The improvement of global NRR could be a comparable occurrence to congenital glaucoma, although we know that such a comparison is controversial. The phenomenon of reversing the glaucomatous cupping of the optic disc following lowering of the IOP was originally recognized in infants [24]. Wu et al. observed a reduction in optic disc cupping after trabeculotomy in primary congenital glaucoma [25]. Similar results were obtained by Meirelles et al., who noted that there was a significant difference between the preoperative and postoperative C/D in childhood glaucoma [26]. One of the mechanisms of cupping reversal seen after lowering IOP in paediatric glaucoma is the shrinkage of a stretched scleral canal [27]. Gietzelt et al. reported that structural reversal of disc cupping after trabeculectomy markedly influences the Bruch membrane opening-based parameters for even more than 1 year [28].

In the current study, none of our patients had side effects after LPI. However, the possibility of postlaser adverse events should be considered before deciding on the laser procedure. Postoperative inflammation, halos, transient hemorrhage, elevated IOP, posterior synechiae, retinal detachment, or cataract may happen [14, 28, 29]. Scott et al. reported 1 case of cataract out of 52 patients after laser treatment in a prospective, randomized, controlled 3-year trial [13]. The most commonly reported adverse event, besides cataract, was mild iritis.

Although the risks of LPI may be minimal, the systematic review by Michelessi and Lindsley found no high-quality evidence for or against the usefulness of iridotomy for improving long‐term outcomes of visual field loss in PG and visual field progression in PDS [14]. A possible reduction in iris concavity and iridozonular contact with less pigment dispersion may not mitigate existing dysfunction of the trabecular meshwork nor lead to a significant reduction in long‐term visual function loss. However, LPI may have long‐term beneficial effects on IOP in eyes with PDS and in eyes at high risk of IOP decompensation. On the other hand, Buffault et al. analyzed randomized controlled trials and two cohort studies (286 eyes of 218 participants), which tried to assess the effects of LPI for PDS and PG [30]. They noticed that the effects of LPI on visual field changes or progression have not been established in PG and PDS. They concluded that there is no scientific evidence to advocate PI as a treatment for PDS and PG.

It is estimated that up to 60–80% of patients with PDS and PG are myopes [7, 31]. Interestingly, increasing myopia is a predictor of increasing iridolenticular contact, independent of the presence of PDS [32]. Campbell suggested that the enlargement of the myopic eye in young patients creates more space for the peripheral iris to bow posteriorly [33]. In the current study, among our 24 patients, more than half of them (14/58, 3%) were myopic and the other 10 had emmetropia at baseline. The results of the last follow-up showed that the number of emmetropic patients has not changed and the percentage of myopic patients was the same as at the start of the study. The degree of myopia has increased in 10 patients (by a mean of −0.83 D), and in 4 patients, their refractive error have remained unchanged. We noticed no significant differences in the effectiveness of LPI in myopic eyes compared with emmetropic eyes. Unfortunately, we omitted the impact of myopia presence on the effect of LPI due to the small sample size.

The main limitation of this study is the small sample size, the COVID-19 pandemic being the main reason. Secondly, there is a relatively short time period of observation of our patients, again due to the pandemic. In the study with a follow-up period of 10–20 years, the relationship between the concave iris configuration and possible PDS development/prevention might be objectifiable. We are aware that additional randomized controlled trials on LPI should be compared to other forms of treatment (or no treatment) in eyes with OHT and iris concavity. We also realize that TonoPen is not the gold standard technique to determine IOP. Furthermore, we do not have a control group, which could consist of patients with one eye after LPI and another with no treatment.

## 5. Conclusion

In the current study, laser peripheral iridotomy resulted in an IOP-lowering effect and iris flattening in adolescent eyes with a concave configuration of the peripheral iris.

## Figures and Tables

**Table 1:** Changes in parameters of the anterior segment: central corneal thickness (CCT), anterior chamber depth (ACD), and scleral spur angle (SSA) before and after the laser treatment.

Parameter on AS-OCT	Before LPI	After LPI	*p* value
CCT-RE (*µ*m)	548 ± 43	551 ± 45	0.565^a^
CCT-LE (*µ*m)	552 ± 42	545 ± 36	0.18^b^
ACD-RE (mm)	3.38 ± 0.29	3.39 ± 0.27	0.41^a^
ACD-LE (mm)	3.35 ± 0.27	3.37 ± 0.22	0.53^a^
SSA in nasal quadrant-RE (°)	54.54 ± 5.62	46.58 ± 7.08	<0.001^a#^
SSA in temporal quadrant-RE (°)	49.29 ± 6.73	44.29 ± 8.6	0.004^a#^
SSA in nasal quadrant-LE (°)	53.42 ± 6.02	48.92 ± 6.04	0.006^a#^
SSA in temporal quadrant-LE (°)	52 ± 6.69	43.63 ± 9.19	<0.001^a#^

^a^ *p* value for the paired *t*-test, ^b^*p* value for the Wilcoxon signed-rank test, and ^#^statistically significant differences at the 0.05 significance level.

**Table 2:** Values of intraocular pressure (IOP) before and after laser peripheral iridotomy.

	Before LPI	After LPI	*p* value
Mean intraocular pressure (IOP) RE (mmHg)	23.21 ± 1.56	17.58 ± 2.64	<0.001^a#^
Mean intraocular pressure (IOP) LE (mmHg)	22.96 ± 2.1	17.58 ± 2.86	<0.001^a#^

^a^ *p* value for the paired *t*-test and ^#^statistically significant differences at the 0.05 significance level.

**Table 3:** Changes in global retinal nerve fiber layer (RNFL) and global neuroretinal rim (NRR) thickness on OCT before and after the laser peripheral iridotomy.

Parameter on SLO/OCT	Before LPI	After LPI	Significance
Global RNFL-RE (*µ*m)	94.63 ± 10.46	96.63 ± 9.64	0.003^b#^
Global RNFL-LE (*µ*m)	95.54 ± 9.6	97.96 ± 11.52	0.07^b^
Global NRR-RE (*µ*)	307.67 ± 50.26	316.75 ± 53.83	0.038^b^
Global NRR-LE (*µ*)	316.92 ± 57. 39	324.63 ± 55.17	0.047^b^

^a^ *p* value for the paired *t*-test and ^#^statistically significant differences at the 0.05 significance level.

**Table 4:** Comparison between gender and IOP reduction after LPI.

	Male (*n* = 14)	Female (*n* = 10)	*p* value^a^
	Mean ± Std	Mean ± Std	
IOP in RE after LPI (mmHg)	16.71 ± 2.23	18.8 ± 2.78	0.07
IOP in LE after LPI (mmHg)	16.86 ± 2.6	18.6 ± 3.03	0.16

^a^ *p* value for the unpaired *t*-test.

**Table 5:** Correlation between age and IOP reduction after LPI.

	Pearson's correlation coefficient	*p* value
IOP in RE after LPI (mmHg)	−0.316	0.13
IOP in LE after LPI (mmHg)	−0.172	0.42

## Data Availability

The statistical data used to support the findings are available from the corresponding author upon request.
